# β-Hydroxybutyrate Attenuates Bisphenol-A-Induced Cellular Responses in Ovarian Cancer Cells

**DOI:** 10.3390/cimb48100995

**Published:** 2026-09-26

**Authors:** Hussein Sakr, Amira Al Kharusi, Fatma Mohamed Fouad, Srinivasa Rao Sirasanagandla

**Affiliations:** 1 Department of Physiology, College of Medicine and Health Sciences, Sultan Qaboos University, Muscat 123, Oman; hsakr@squ.edu.om (H.S.); akharusi@squ.edu.om (A.A.K.); fatmamohamed548@gmail.com (F.M.F.); 2 Department of Human and Clinical Anatomy College of Medicine and Health Sciences, Sultan Qaboos University, Muscat 123, Oman

**Keywords:** bisphenol-A, β-hydroxybutyrate, glucose, wound healing, signaling pathways, ovarian cancer

## Abstract

Bisphenol A (BPA) is an endocrine-disrupting chemical that is widely known to modulate cancer-related cellular processes, while β-hydroxybutyrate (BHB), a ketone body, has been implicated in regulating cancer cell metabolism and signaling. The interaction between BPA and BHB under glucose-controlled conditions in Ovarian cancer (OC) cells is not well studied. The OC cell line (SKOV-3) was treated with BHB, BPA, or their combination in the presence or absence of glucose. Cell viability and wound-healing ability were assessed using Alamar Blue dye and wound-healing assays, respectively. Also, the expression of estrogen receptors (*Erα* and *ERβ*), the apoptotic marker (*caspase-3*), *PIK3CA*, *AKT1*, and epithelial–mesenchymal transition (EMT) markers (*CDH1* and *CDH2*) was monitored using qPCR. Additionally, common genes between BPA, BHB, and OC were checked through online tools such as Swiss Target Prediction and the ChEMBL database for BPA and BHB’s target genes, and GeneCards, OMIM, and Comparative Toxicogenomics Database (CTD) were used to detect target genes in OC. Then, a protein–protein Interaction (PPI) Network was constructed from shared genes. Gene Ontology (GO) and KEGG were detected between common genes through the STRING database. BHB (5 mM) treatment significantly inhibited the BPA (1 μM) exposure-induced increase in SKOV-3 cell viability (*p* < 0.05), under glucose-controlled conditions. The combined treatment of BPA and BHB significantly reduced wound closure compared with the BPA-treated group alone, both in the presence/absence of glucose (*p* < 0.05). The expression of *Erα* (*ESR1*), *Caspase-3*, *PIK3CA*, *AKT1*, and *CDH1* was increased in the BPA+BHB—glucose-group while decreased in BPA-treated cells alone. However, *CDH2* showed decrease in the BPA+BHB—glucose-group compared with BPA group. According to our bioinformatics analysis, eighteen genes were common between BPA, BHB, and OC. Among these genes, *HMGCR*, *PPARG*, *HDAC6*, and *ESR2* were the top four target genes. Activation of cysteine-type endopeptidase activity involved in the apoptotic process was the top GO term, and osteoclast differentiation was detected as the enriched KEGG pathway among the shared genes. These findings suggest that BPA promotes SKOV 3 cell viability and wound-healing ability, while BHB altered BPA-induced cellular responses in a glucose-dependent condition and is linked with changes in estrogen receptor, apoptotic, PI3K/AKT1, and EMT-related gene expression. These results demonstrate that the cellular responses to combined BPA and BHB treatment in OC cells may be affected by metabolic conditions.

## 1. Introduction

Ovarian cancer (OC) is the deadliest gynecological malignancy, with a high probability of recurrence, reaching up to 75% [1,2]. The majority of OC cases are classified as epithelial malignancies, with high-grade serous ovarian cancer accounting for 70–80% of these cases [1]. High-grade serous ovarian cancer is usually identified at an advanced stage, contributing to its high mortality rate [3,4]. OC generally has a poor prognosis partly due to its asymptomatic nature in the early stages, which often results in late presentation [5]. The five-year survival rate for OC patients at advanced stages is only 17% [3]. Therefore, understanding the mechanisms of OC progression and identifying key risk factors are essential for improving survival outcomes.

Bisphenol A (BPA) is a monomer that is commonly used in commercial products such as plastics, food containers, and coating materials for teeth [6,7]. BPA can be inhaled, ingested with food, and absorbed through the skin of humans [6]. Moreover, BPA has been detected in human urine samples in a range from 0.005 to 2.7 ng/mL [8], and from 0–1.6 ng/mL in serum samples [7]. Several studies have proven the negative side effects of low concentrations of BPA on health [9]. BPA is widely known to disrupt the endocrine system [6,7]. When the human body is exposed to BPA, the liver starts the metabolism and absorption of BPA; which is then transferred to the blood, where plasma proteins bind to BPA and facilitate its transport to several tissues, particularly reproductive tissues [9]. BPA impacts the female reproductive system as it induces the production of multi-oocyte follicles [9,10]. Moreover, BPA can cause an imbalance in ovarian hormone levels, such as estradiol, progesterone, testosterone, and prolactin [9,11]. BPA is an environmental estrogen that can interact with estrogen receptors and produce a similar effect to estrogen [12].

Increasing evidence suggests that BPA contributes to cancer progression through different molecular mechanisms. BPA in low concentrations increase the cell viability of thyroid cancer through interaction with the estrogen membrane receptors that induce the AKT/mTOR pathway [13]. Also, BPA can promote neoplastic transformation in epithelial cells of the breast, in addition to inducing cell proliferation [14,15] and migration in cancer cells [14]. In OC, BPA depends on estrogen signaling pathways to induce cell proliferation, migration, and invasion [16]. *MMP-2* and *MMP-9* genes and the PI3K-AKT signaling pathway are overexpressed in OC cells after exposure to BPA, and this expression is reduced when an ERα inhibitor is used, alongside a reduction in metastasis in OC [16,17]. Therefore, BPA has been documented to induce metastasis in OC through the PI3K-AKT signaling pathway. In a recent study, BPF has been shown to be involved in ovarian carcinogenesis by promoting SKOV-3 cells proliferation and migration [18]. Targeting cancer metabolism has emerged as a promising therapeutic strategy. Because cancer cells depend on aerobic glycolysis to metabolize glucose for energy production [19], ketogenic diets have been investigated in some cancer types as a means of restricting glucose availability and inhibiting cancer progression [20,21]. Earlier studies have demonstrated the beneficial effects of natural compounds or natural products in mitigating BPA exposure-induced toxicity, by acting through their anti-inflammatory, antioxidant and cytoprotective properties [22]. Beta-hydroxybutyrate (BHB) is a ketone body that is produced during a ketogenic diet (KD). It has several roles in cellular metabolism, as it is considered a source of energy [23]. In some types of cancer, BHB increases apoptosis and decreases cell proliferation and migration [24,25,26]. To date, the effects of BHB on BPA-treated OC cells have not been studied. Therefore, in this study, we aimed to evaluate the effect of low concentrations of BPA on OC cells, and to determine whether BHB modulates these effects in the presence and absence of glucose.

## 2. Materials and Methods

### 2.1. Chemicals and Reagents

BPA (Sigma Aldrich, USA, #239658) was dissolved in 99.9% dimethyl sulfoxide (DMSO) (Sigma-Aldrich, St. Louis, MO, USA), and 0.4 M of BPA was used. The final DMSO concentration was kept at 0.1%, with a 0.1% DMSO-treated medium serving as the control. BHB (TRC; Toronto, ON, Canada) was dissolved in dH_2_O to prepare 0.4 M stock.

### 2.2. Cell Culture

The non-HGSOC (High-Grade Serous Ovarian Cancer) cell line, SKOV-3 was used. The SKOV-3 cell line was kindly provided by Professor Aikou Okamoto from Jikei University School of Medicine, Tokyo, Japan. The SKOV-3 cell line is an established human ovarian cancer cell line that has been previously described in the literature [24]. Cells were cultured in RPMI media (Gibco™, Thermo Fisher Scientific, Waltham, MA, USA; Cat. No. A1049101) with 10% FBS (Gibco™, Thermo Fisher Scientific, Waltham, MA, USA; Cat. No. A5256701) and 1% Penicillin-Streptomycin (Thermo Fisher Scientific, Waltham, MA, USA; Cat. No. 15140122), and incubated at 37 °C under 5% CO_2_.

### 2.3. Cell Viability Assay

SKOV-3 cells (7 × 10^3^) were cultured in 96-well plates for 24 h. Two concentrations of BHB (5 mM, and 20 mM) were added and the cells were propagated for 24 h. Subsequently, the media was replaced with 90 µL of fresh RPMI containing 10% FBS and 10 µL of Alamar blue (Invitrogen™, Thermo Fisher Scientific, Waltham, MA, USA; Cat. No. DAL1025), and the cells were incubated for 4 h.

In a parallel experiment, different concentrations of BPA ranging from 0.0001 to 1 µM were added to cells cultured in a 96-well plate. Based on the optimization, 1 µM BPA was selected for further experiments.

Subsequently, the following four treatment groups were included: control (DMSO), 1 µM BPA alone (BPA 1 µM), 1 µM BPA with BHB and RPMI containing 10% FBS and 25 mM glucose (BPA 1 µM+BHB+G), and 1 µM BPA with BHB and media with 10% FBS and 0 mM glucose (BPA 1 µM+BHB-G). BPA was added for 24 h, then BHB and media with 25 mM or 0 mM glucose were administered for another 24 h. Then, media was removed, and 90 µL of fresh RPM1 with 10% FBS and 25 mM glucose media and 10 µL of Alamar blue reagent were added to the 96-well plates. The plates were read at 570 nm after four hours of incubation.

### 2.4. Wound-Healing Assay

SKOV-3 cells (2 × 10^5^) were seeded in a six-well plate until they reached 80% confluency. Using a 200 µL sterile tip, a wound was created in the middle of each well. The cells were treated with DMSO (control), BPA (1 µM), BPA (1 µM)+BHB+glucose, and BPA (1 µM)+BHB—glucose, and pictures of the wound were taken every day for three days using a Nikon Eclipse Ti2 S microscope (Nikon Corporation, Yokohama, Japan) at a 4× objective lens. ImageJ software (version 1.54g; National Institutes of Health, Bethesda, MD, USA) was used to measure the area of the wound. This experiment was repeated three times independently.

### 2.5. Real-Time PCR (qPCR)

Total RNA was extracted from treated cells using TRIzol Reagent (Invitrogen, Carlsbad, CA, USA; Cat. No. 15596026). RNA was converted to cDNA using a high-capacity reverse transcription kit (Applied Biosystems™, Thermo Fisher Scientific, Waltham, MA, USA; Cat. No. 4374966). We used SYBR Green PCR Master Mix (Applied Biosystems™, Thermo Fisher Scientific, Waltham, MA, USA; Cat. No. 4309155), 15 ng of cDNA, and 100 μM of primers (Table 1) to perform a qPCR reaction. Gene expression was detected using the 7500 Fast Real-time PCR system (Applied Biosystems, Waltham, MA, USA). The 2^−ΔΔCT^ method was used to analyze the data. Each experiment was performed in three independent replicates. Estrogen receptor signaling markers (*Erα/β*), cell survival, proliferation, and metabolism markers (*PI3K* and *AKT*), epithelial–mesenchymal transition markers (*CDH1: E-cadherin*; and *CDH2: N-cadherin*), and the pro-apoptotic marker (*Caspase 3*) were selected for analysis in SKOV-3 cells after treatment with BPA alone or BPA+BHB with/without glucose. *β-actin* was used as housekeeping gene.

### 2.6. Identification of BPA, BHB, and OC Target Genes

BPA and BHB-related genes were collected from Swiss Target Prediction (http://www.swisstargetprediction.ch/) (accessed on 9 August 2026) and the ChEMBL database (https://www.ebi.ac.uk/chembl/) (accessed on 9 August 2026). The SMILES strings of BPA (CC(C)(C1=CC=C(O)C=C1)C1=CC=C(O)C=C1) and BHB (CC(O)CC([O-])=O) were taken from DrugBank (https://go.drugbank.com/) (accessed on 9 August 2026). Genes were selected from Swiss Target Prediction according to a probability ≥ 0.10 and for “Homo sapiens”. The Homo sapiens genes from ChEMBL database were then compared with those from Swiss Target Prediction, and repeated genes were removed. GeneCards database (https://www.genecards.org/) (accessed on 10 August 2026), OMIM (https://omim.org/) (accessed on 10 August 2026), and Comparative Toxicogenomics Database (CTD) (https://ctdbase.org/) (accessed on 10 August 2026) were used to find genes related to OC. Then, any duplicated genes among the resulting datasets were removed.

### 2.7. Establishment of Venn Diagram and Protein–Protein Interaction (PPI) Network

We used the Venny 2.1 website (https://bioinfogp.cnb.csic.es/tools/venny/) (accessed on 10 August 2026) to construct a venn diagram and select the common genes between BPA, BHB, and OC. Then, the STRING database online (https://string-db.org/) (accessed on 10 August 2026) was used to design the protein–protein interaction (PPI) network of shared genes between BPA, BHB, and OC. Analysis parameters were set to “Homo sapiens”, and we used a minimum interaction score of >0.1 (low confidence level). Gene ontology (GO) and KEGG pathway analyses of these common genes were checked using the online STRING database. The results from STRING were transferred to Cytoscape software (version 3.10.4) to get a graphical visualization and assess PPI network topology. The statistical parameters of the PPI network, such as betweenness centrality, closeness centrality, and degree, were measured to confirm the central nodes with high connectivity within the network.

### 2.8. Statistical Analysis

Data were analyzed by SPSS software version 21 (IBM Corp., Armonk, NY, USA) and represented using GraphPad Prism software, version 8.0.2 (GraphPad Software, San Diego, CA, USA). Data were presented as the mean ± SEM of three independent biological replicates. One-way analysis of variance (ANOVA), followed by Fisher’s least significant difference (LSD) post hoc test, was used to compare differences among groups. A value of *p* < 0.05 was considered statistically significant.

## 3. Results

### 3.1. Effects of BHB on Cell Viability

To determine the effects of BHB on OC cell growth, the viability of SKOV-3 cells was assessed following exposure to two different BHB concentrations (5 mM and 20 mM) using Alamar blue reagent. Both 5 mM and 20 mM BHB significantly reduced SKOV-3 cell viability compared to the control, suggesting an inhibitory effect of BHB on cell viability. The decrease in cell viability was more pronounced in cells treated with 5 mM (83.4%) than 20 mM (85.7%) (*p* < 0.05; Figure 1A). Hence, 5 mM BHB was used for further experiments.

### 3.2. Effects of BPA on Cell Viability

To determine the effects of BPA on OC cell growth, the viability of SKOV-3 cells was assessed following exposure to a range of BPA concentrations (0.0001–1 µM) using Alamar blue reagent for 24 h. As shown in Figure 1B, cell viability was higher in cells treated with 1 µM BPA compared to the control (149.6%, *p* < 0.05), suggesting potential stimulatory effects on cell viability. Hence, 1 µM BPA was used in subsequent experiments.

### 3.3. Effect of BPA, BHB and Glucose Concentration on SKOV-3 Cell Viability

To analyze the combined effects of BPA, BHB, and glucose availability on SKOV-3 cell viability, the optimized BHB concentration (5 mM) was used in subsequent experiments with 1 µM BPA. SKOV-3 cells were treated with 1 µM BPA and 5 mM BHB in the presence and absence of glucose. This combination of the two treatments did not show any significant difference in cell viability in the presence or absence of glucose (Figure 1C) (*p* > 0.05).

### 3.4. Effect of BPA, BHB and Glucose Concentration on SKOV-3 Cell Morphology

As indicated in Figure 2, no significant change in nuclear length was observed in BPA-treated SKOV-3 cells compared with the control (*p* > 0.05). In contrast, nuclear shrinkage was observed in both BPA+BHB + glucose and BPA+BHB—glucose groups, compared with the control (*p* < 0.001 and *p* < 0.01 respectively), and the BPA-treated group (*p* < 0.001 and *p* < 0.001 respectively).

### 3.5. Effect of BPA, BHB in the Presence and Absence of Glucose on Wound-Healing Ability

The effects of BPA and BHB on migration ability of SKOV-3 cells were investigated in the presence and absence of glucose. Cells were grown in six-well plates, propagated overnight, then treated with DMSO (control), 1 µM BPA, and 5 mM BHB in the presence and absence of glucose. Wound-healing ability was monitored at 0 h, 24 h, and 48 h. As shown in Figure 3, a decrease in wound closure was observed in both the 1 µM BPA+BHB-G and 1 µM BPA+BHB+G treated groups (47%, *p* < 0.01 and 57% *p* < 0.05, respectively) compared to the 1 µM BPA treated group, while the 1 µM BPA treatment group showed an increase in wound-repair ability (70%; *p* < 0.05) compared to the control (57.2%). These findings suggest that BHB attenuates the pro-migratory effect of BPA.

### 3.6. Effect of BPA and BHB on mRNA Expression in SKOV-3 Cells in the Presence and Absence of Glucose

The mRNA expression of *Erα*, *Erβ*, *Caspase 3*, *PIK3CA*, *AKT1*, *CDH1*, and *CDH2* was assessed to evaluate the effects of BPA and BHB on estrogen receptor signaling, cell survival, and proliferative pathways, epithelial–mesenchymal transition, and apoptosis in SKOV-3 cells under the presence/absence of glucose. The expression of *Erα* was higher in both the 1 µM BPA+BHB+G and 1 µM BPA+BHB-G groups compared to the control (*p* < 0.01 and *p* < 0.05) and BPA alone (*p* < 0.01; Figure 4A). The expression of *Erβ* and *PIK3CA* increased in the 1 µM BPA+BHB-G group compared to the control (*p* < 0.05 and *p* < 0.01, respectively) (Figure 4B,D). Additionally, the levels of *AKT1* and *PIK3CA* up regulated in the group 1 µM BPA+BHB-G compared to the 1 µM BPA group (*p* < 0.05, *p* < 0.01) (Figure 4E,D). Moreover, the proapoptotic marker, *Caspase 3*, was overexpressed in the 1 µM BPA+BHB-G group compared to control and 1 µM BPA (*p* < 0.05, *p* < 0.01); additionally, it was highly regulated in the 1 µM BPA+BHB+G group when compared with the 1 µM BPA group (*p* < 0.05) (Figure 4C). Moreover, *CDH1* was up regulated in the 1 µM BPA+BHB-G groups in comparison with the 1 µM BPA group (*p* < 0.05) (Figure 4F), while *CDH2* expression was downregulated in 1 µM BPA+BHB-G group compared with the 1 µM BPA group (*p* < 0.05, Figure 4G).

### 3.7. Identification of Shared Genes Between BPA, BHB, and OC

Through the SwissTargetPrediction and ChEMBL databases, we found 155 target genes for BPA and 101 for BHB with a probability ≥ 0.10. However, 11,963 genes were found as target genes of OC in the GeneCards database, OMIM, and the Comparative Toxicogenomics Database. As shown in the Venn diagram (Figure 5), 18 genes were common between BPA, BHB, and OC.

### 3.8. Protein–Protein Interaction Network Analysis

Eighteen genes that were common between BPA, BHB and OC were imported into the STRING database, and 15 of these were successfully mapped to form an interaction network (Figure 6). Node size and color intensity were scaled proportionally to reflect the degree values of the targets, using a yellow to dark blue gradient. Genes with dark blue nodes and larger sizes have the most important role in the interaction network, with *PPARG* identified as the most important gene.

### 3.9. GO and KEGG Pathway Enrichment Analysis

The GO analysis of the 18 genes was explored through the STRING database. The activation of cysteine-type endopeptidase activity involved in the apoptotic process and Positive regulation of cysteine-type endopeptidase activity were the top biological functions of these genes (Figure 7A). In the molecular function category, zinc ion binding appears as the most prominent function, whereas i on binding has the lowest FDR and the highest gene count (Figure 7B). For KEGG pathways, only two pathways were related to these genes: nitrogen metabolism and osteoclast differentiation (Figure 7C).

## 4. Discussion

In this study, we investigated the effect of BHB on SKOV-3 cells treated with BPA in the presence and absence of glucose. Importantly, our findings demonstrate that BHB and BPA may exert opposing effects on SKOV 3 ovarian cancer cells, highlighting their distinct roles in influencing cellular responses under glucose-controlled conditions.

In the present study, BPA enhanced SKOV-3 cell viability, with a significant increase observed at the higher concentration (1 μM). Several studies have reported the harmful effects of BPA on human health [27]. In most types of hormone-associated cancer, BPA has been reported to induce cancer progression, migration, and invasion, as it can resemble estrogen and bind to estrogen receptors [28]. Consistent with our findings, BPA increased cell viability in OVCAR-3 and ES-2 with increasing BPA concentrations [16,17]. In contrast to our results, Yuan et al. observed that 1μM BPA had a non-significant effect on SKOV-3 cell viability [29]. Similarly, in some breast cancer cells, cell viability did not change with the BPA concentration [30].

In contrast to BPA, 5 mM BHB significantly decreased SKOV 3 cell viability in our study. Our findings are in agreement with previous observations that BHB can impart inhibitory effects on OC cells. Ghahremani et al. (2021) reported that BHB inhibited cell proliferation of SKOV-3 and A2780CP ovarian cancer cells at concentrations above 5 mM [31]. Similarly, Miller et al. (2023) observed a decrease in SKOV 3 cell proliferation when treated with approximately 40–60 mM of BHB [32]. Taken together, the opposing effects of BPA and BHB on SKOV-3 cell viability suggest that these compounds could differentially regulate cellular responses. Under normal physiological conditions, serum BHB concentrations are generally within the low micromolar range. These concentrations increase to several hundred micromolar after 12–16 h of fasting, rise to 1–2 mM after approximately 2 days of fasting, and can reach 6–8 mM during long-lasting starvation [33]. Glucose is a crucial energy substrate for cancer cells, while BHB can serve as an alternative metabolic fuel [34]. Hence, alterations in glucose availability could affect the responses of cancer cells to BHB and other metabolic interventions, such as a KD. The KD forces cancer cells to use oxidative phosphorylation instead of glycolysis for ATP production [35]. When some researchers studied the effect of a KD on neuroblastoma cell lines that were treated with chemotherapy, the KD decreased the growth of the tumor and extended the survival rate in a mouse model [36]. On the other hand, in an epithelial ovarian cancer mouse model, a KD with unrestricted food intake induced tumor progression [37]. Interestingly, in our study, the combination of BPA+BHB treatment resulted in no significant difference in cell viability in comparison to the control group in both glucose-containing and glucose-free conditions. This result suggests that BHB may compensate for the BPA-induced increase in SKOV 3 cell viability, thereby attenuating the proliferative effects of BPA. Hence, BHB could alter the cellular response to BPA, resulting in an overall effect that differs from that observed with either treatment individually.

According to previous research, BPA increased the tumor size in mice implanted with BG-1 ovarian cancer cells [38]. However, BHB reduced tumor volume in EOC mouse models after 16-h intermittent fasting [39]. Consistent with these previous findings, our study demonstrated that the combination of BPA+BHB+/− glucose treatment decreased the nuclear size of SKOV-3 cells.

In terms of wound-healing capacity, BPA enhanced wound closure in SKOV 3 cells in comparison to the control, while gap closure was reduced in BPA+BHB+glucose and BPA+BHB—glucose-treated groups. These results suggest that BHB can reduce BPA-associated wound closure in SKOV-3 cells and that these effects are consistent in the presence or absence of glucose. In agreement with our findings, BPA enhanced the migration of OVCAR-3 OC cells by activating MAPK and PI3K/Akt signaling cascades [40]. In contrast, evidence regarding BHB’s effect on cancer cell migration is less consistent. Bonuccelli et al. (2010) reported that BHB promotes tumor growth and metastasis in breast cancer models without significantly increasing tumor angiogenesis [41]. However, a study on A2780CP ovarian cancer cells showed that BHB did not influence migration in glucose-restricted conditions in short-term experiments [42].

Furthermore, the expression of estrogen receptors (*Erα* and *ERβ*), apoptotic marker (*caspase-3*), *PIK3CA*, *AKT1*, and epithelial–mesenchymal transition (EMT) markers (*CDH1* and *CDH2*) was assessed to investigate the effects of BHB and BPA on estrogen receptor signaling, cell survival, apoptosis, and EMT. Erα is known to have an oncogenic role and promote tumor growth and progression [43]. The upregulation in *Erα* expression post-BHB treatment may not necessarily represent enhanced functional Erα signaling, since a previous study has reported alterations in *Erα* in SKOV 3 cells [44]. Hence, increased *Erα* levels after BHB treatment may represent a compensatory response or altered modulation of the receptor instead of pro-proliferative effects. The expression of *ERβ* was highest in the BPA+BHB—glucose group compared with the control. ERβ is known to function as a tumor suppressor in OC, and it inhibits cell growth and invasiveness [45]. The significant rise in *ERβ* expression suggests that glucose unavailability could affect the molecular response to BHB and its association with ER signaling. Hence, BHB could influence the balance of ER signaling, as indicated by the changes in *ERβ* levels.

The increase in *caspase-3* expression in both BPA+BHB groups with and without glucose suggests that BHB may enhance apoptotic signaling in BPA-treated OC cells. This is consistent with the published literature, including studies on non-small cell lung cancer, in which BHB increased cleaved caspase-3 levels and promoted apoptosis [46]. Similarly, BHB improved oxaliplatin sensitivity in colorectal cancer by upregulating cleaved caspase-3 levels in vivo [47]. The observed increase in caspase-3 expression is consistent with our bioinformatics findings, which indicated activation of cysteine-type endopeptidase activity involved under the apoptotic process in the biological process in GO and the osteoclast differentiation pathway. Previous studies have documented a role for caspase-3 activity in RANKL induced osteoclast differentiation [48]. The PI3K/AKT signaling pathway is a crucial regulator of cell proliferation, survival, and metabolism and is commonly altered in OC [49]. However, in our study, PI3K/AKT was upregulated in BPA+BHB groups without glucose compared with the BPA group. SKOV-3 cells treated with BPA+BHB with and without glucose cells may use PI3K/AKT in a compensatory survival response. Our analysis of the expression of EMT markers demonstrated up-regulation of *CDH1* and down-regulation of *CDH2* expression in BPA+BHB—glucose treated SKOV 3 cells compared to BPA-treated cells. On the other side, a previous study confirmed the decrease in *CDH1* and increase in *CDH2* in OC after exposure to BPA [50]. The increase in wound closure following BPA treatment is consistent with the downregulation of *CDH2* and increased *CDH1*, supporting an EMT-associated migratory phenotype in SKOV-3 cells. Moreover, BHB significantly inhibited wound closure compared to the BPA-treated group without glucose, by restoring *CDH1* levels, suggesting that BHB could partially attenuate BPA-induced EMT-related alterations.

To further validate our findings and elucidate the potential molecular mechanisms underlying the effects of BPA and BHB in OC, bioinformatics analyses were conducted to identify key hub genes, PPIs, and relevant biological pathways that could modulate the observed cellular responses. In our bioinformatics study, eighteen genes have been found as potential targets for BPA, BHB, and OC through network toxicology analysis. *HMGCR*, *PPARG*, *HDAC6*, and *ESR2* were the top four predicted target genes by degree. *HMGCR* is an important gene in the androgen and cholesterol synthesis pathway [51,52]. It was found to be one of the highest-ranked hub genes between bisphenol B, analog of BPA and OC [51]. Further, BPA inhibits the expression of *PPARG* gene in ovary tissue [53]. It regulates lipid metabolism in adipose tissue and liver [54]. On the contrary, *PPARG* was up regulated in skeletal muscle and liver in male mice after exposure to a keto diet and BHB [55]. Moreover, the increase in HDAC6 expression induces epithelial–mesenchymal transition (EMT), cell invasion, and migration [56]. The exposure of rats to BPA induces the expression of HDAC6 and suppresses the PTEN tumor suppressor gene, and activates phosphorylated Akt and the Myc-oncogene [56]. Among the identified hub genes, ESR2 is one of nuclear estrogen receptors that bind estrogen and regulate estradiol (E2) function [57]. When female rats were exposed to BPA for 90 days, the expression of *ESR2* did not change [57]. However, the expression of *ESR2* increased in MCF7 breast cancer cells, when treated with BPA for 15 days [58]. Rana et al. (2025) observed that *ESR2* expression was inhibited in female mice with depletion of gonadal hormones after keto-diet feeding [59]. The shared genes predicted through our bioinformatic analysis represent potential candidate genes associated with the response to the BPA and BHB combination. However, these findings require further experimental validation to understand their specific roles and whether they directly contribute to the observed effects.

Hence, BHB has the potential to reprogram the response to BPA-induced effects in SKOV 3 cells in a metabolically dependent manner, promoting apoptotic signaling and inducing epithelial-associated *CDH1* expression, while modifying estrogen receptor and activating PI3K/AKT signaling. These results provide insight into the molecular mechanism of action of BPA and BHB in SKOV 3 cells under glucose-controlled conditions.

The present study has the following limitations. The study was conducted using only one ovarian cancer cell line, SKOV-3, which may not fully represent the heterogeneity of OC. Conducting in vivo studies using animal models of OC is warranted to further validate the findings of the study.

## 5. Conclusions

In conclusion, BPA increased SKOV-3 cell viability and wound closure, indicating pro-survival effects and increased migratory effects in OC. In contrast, BHB decreased cell viability and attenuated BPA-induced migratory behavior in both glucose-containing and glucose-free conditions. Hence, BHB in the presence and absence of glucose may counteract some of the cellular responses associated with BPA in OC. Moreover, combining BPA and BHB treatment altered the expression of estrogen receptors, *caspase-3*, *PI3KCA*, *AKT1*, *CDH1*, and *CDH2*, with several of these responses depending on glucose availability. Furthermore, eighteen genes were detected as potential targets for BPA and BHB in OC. Most of the genes have roles in the activation of cysteine-type endopeptidase activity involved in the apoptotic process and osteoclast differentiation pathway. Therefore, the combination of BPA and BHB may be associated with apoptosis-related signaling in OC. Additionally, these findings suggest that BHB could potentially regulate BPA-associated OC cell responses through estrogen signaling, PI3K/AKT signaling, and EMT-related genes according to glucose levels. Additional studies are required to determine the mechanisms underlying these effects.

## Figures and Tables

**Figure 1:**
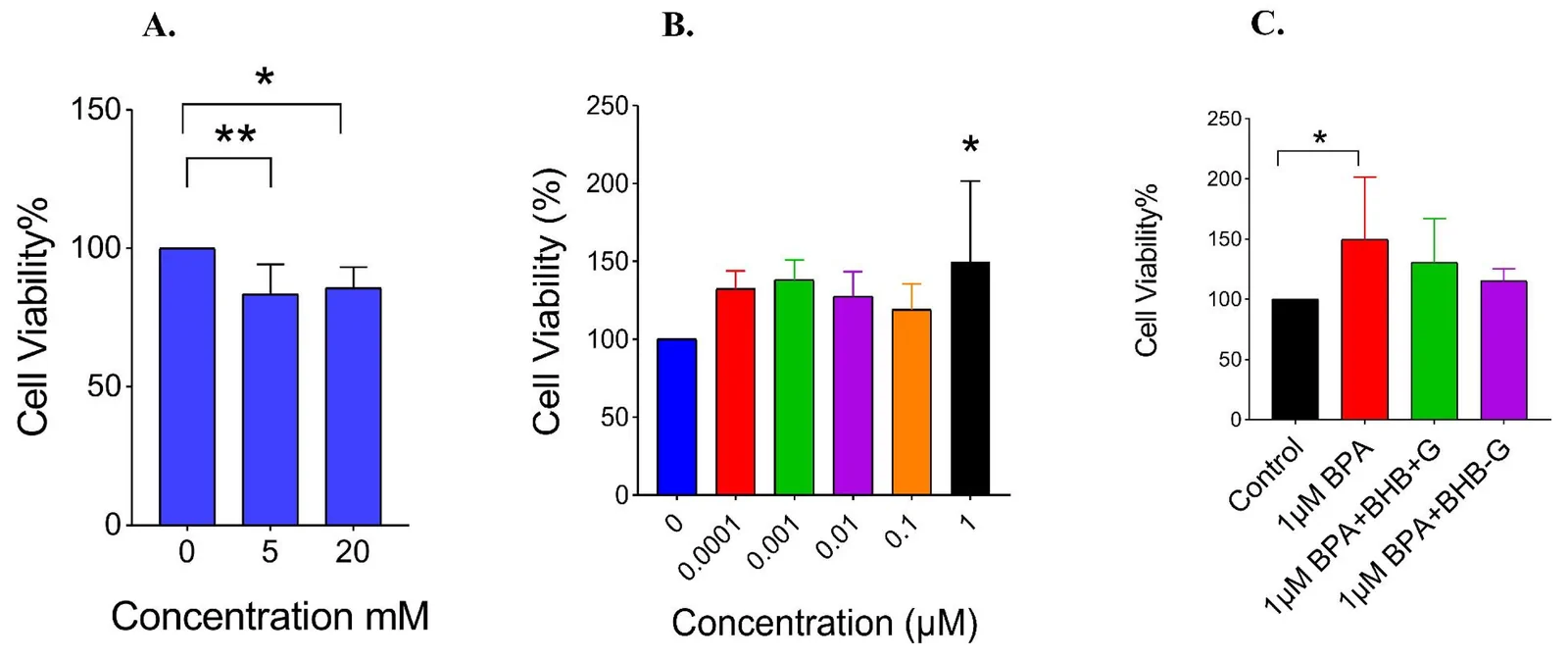
The effect of BPA, BHB, and combination treatments on cell viability of SKOV-3 cells. (**A**) Cell proliferation percentage after exposure to BHB. (**B**) Cell proliferation percentage after exposure to different concentrations of BPA cells. (**C**) Cell proliferation percentage after exposure to a combination of BPA and BHB on cell proliferation in SKOV-3 ovarian cancer cells. *p* ≥ 0.05; *: *p* < 0.05; **: *p* < 0.01.

**Figure 2:**
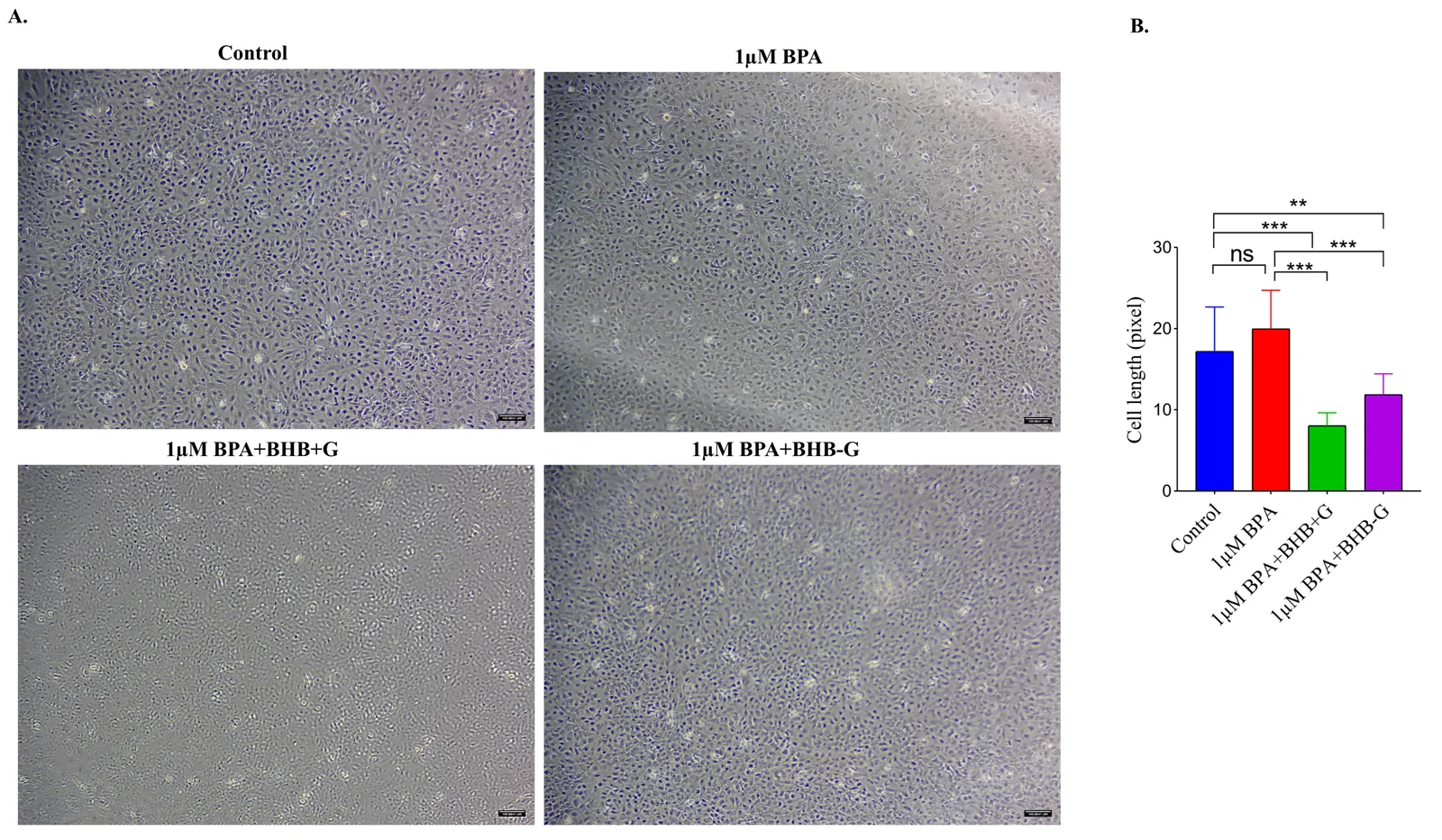
Cell morphology of SKOV-3 cells after treatment. (**A**) Microscopic images of SKOV-3 cells captured using a 4X cell objective. (**B**) Quantification of nuclear length in SKOV-3 cells using ImageJ 1.54g (pixels). ns: not significant; *p* ≥ 0.05; **: *p* < 0.01; ***: *p* < 0.001.

**Figure 3:**
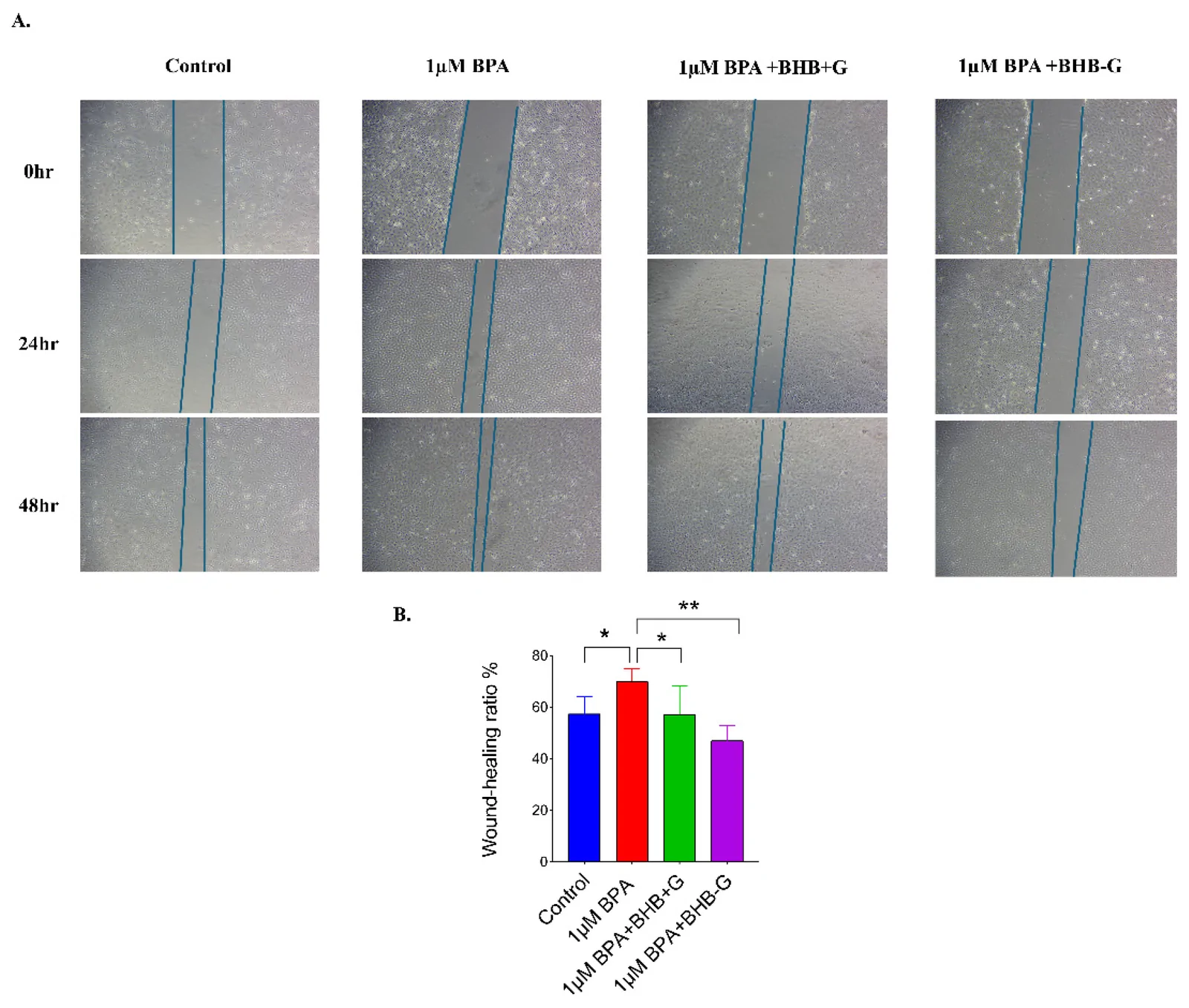
The effect of a combination of BPA and BHB on wound-healing ability in SKOV-3 ovarian cancer cells. The images were obtained using a 4× objective. (**A**) Microscopic images of wound closure were obtained using a 4× objective, and a 100 μm scale bar. (**B**) Quantification of the distance of wound closure using ImageJ software version 1.54g. *p* ≥ 0.05; *: *p* < 0.05; **: *p* < 0.01.

**Figure 4:**
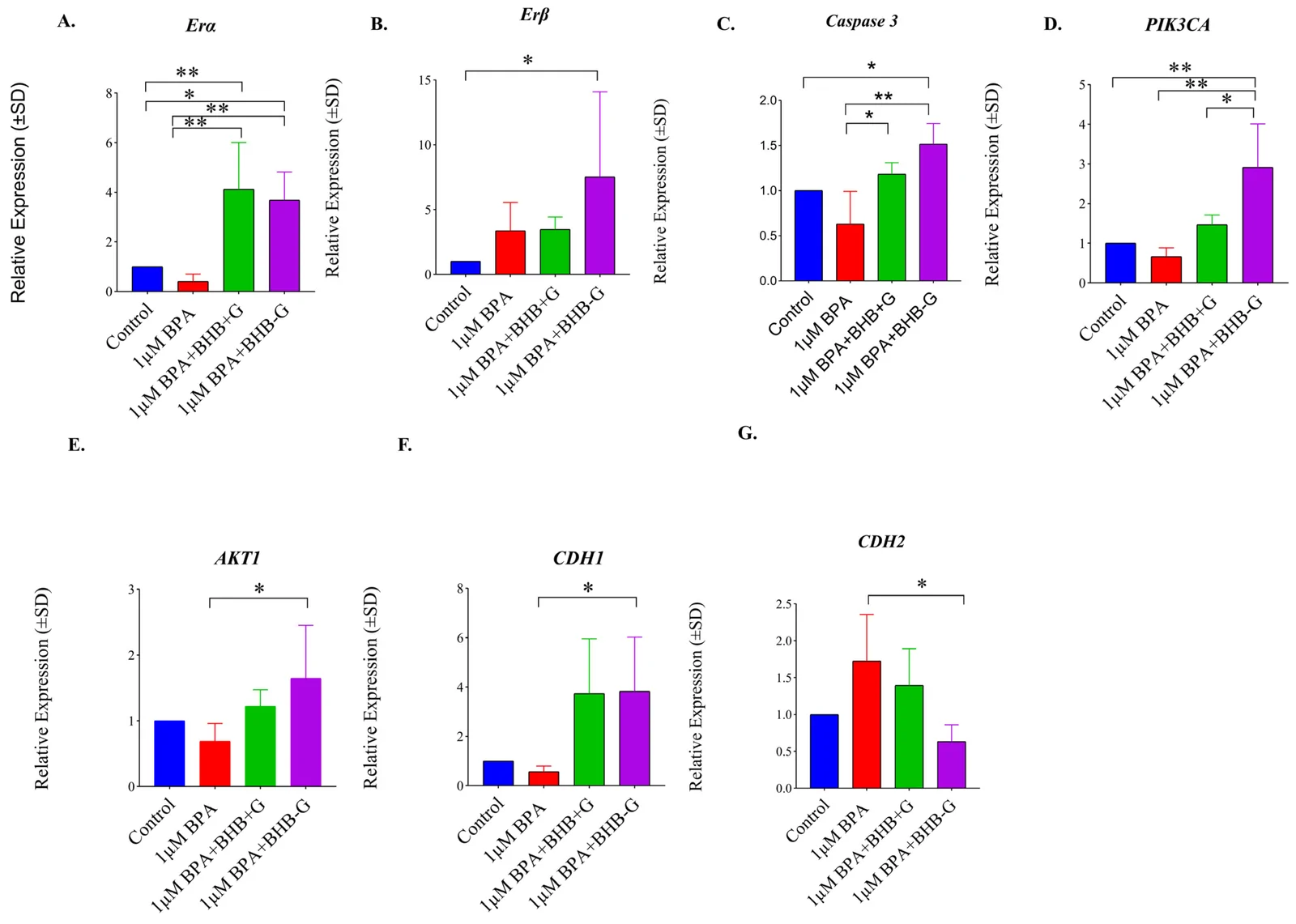
The effect of a combination of BPA and BHB on gene expression in SKOV-3 ovarian cancer cells. (**A**) *Erα*, (**B**) Er*β*, (**C**) *Caspase 3*, (**D**) *PIK3CA*, (**E**) *AKT1*, (**F**) *CDH1*, and (**G**) *CDH2*. *p* ≥ 0.05; *: *p* < 0.05; **: *p* < 0.01.

**Figure 5:**
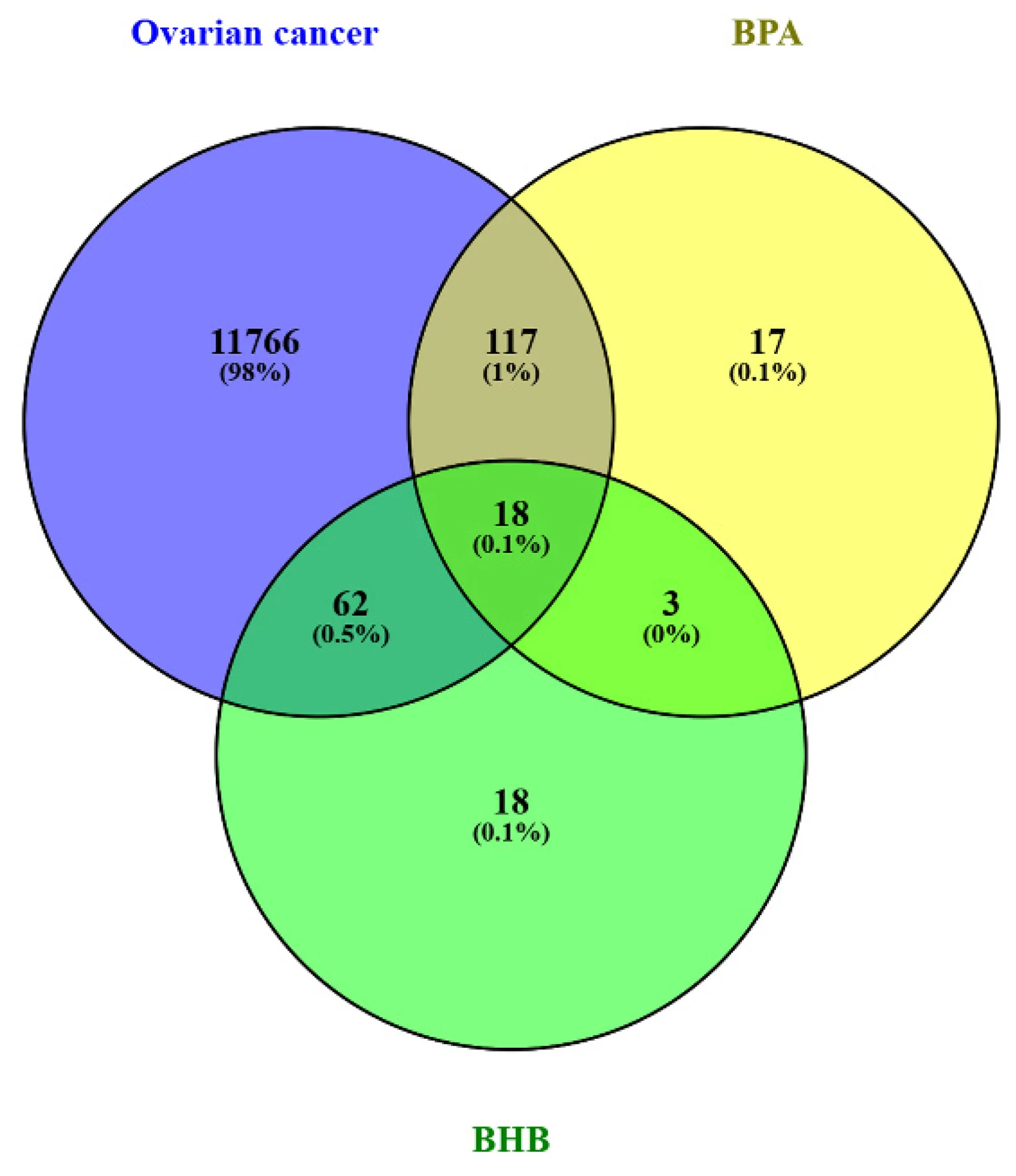
Venn diagram of the shared genes between BPA, BHB, and OC.

**Figure 6:**
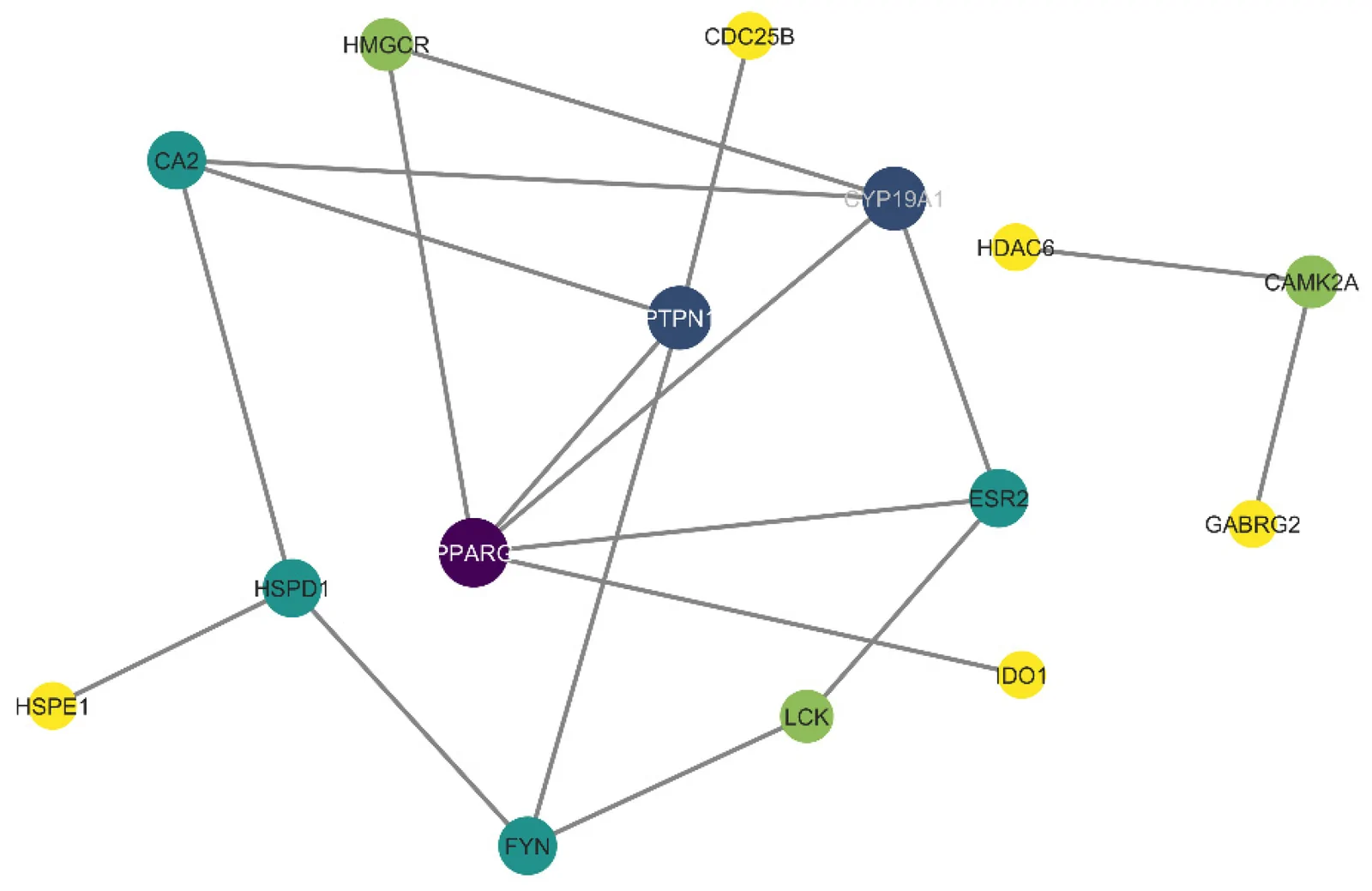
Protein–protein interactions of the common genes between BPA, BHB, and OC.

**Figure 7:**
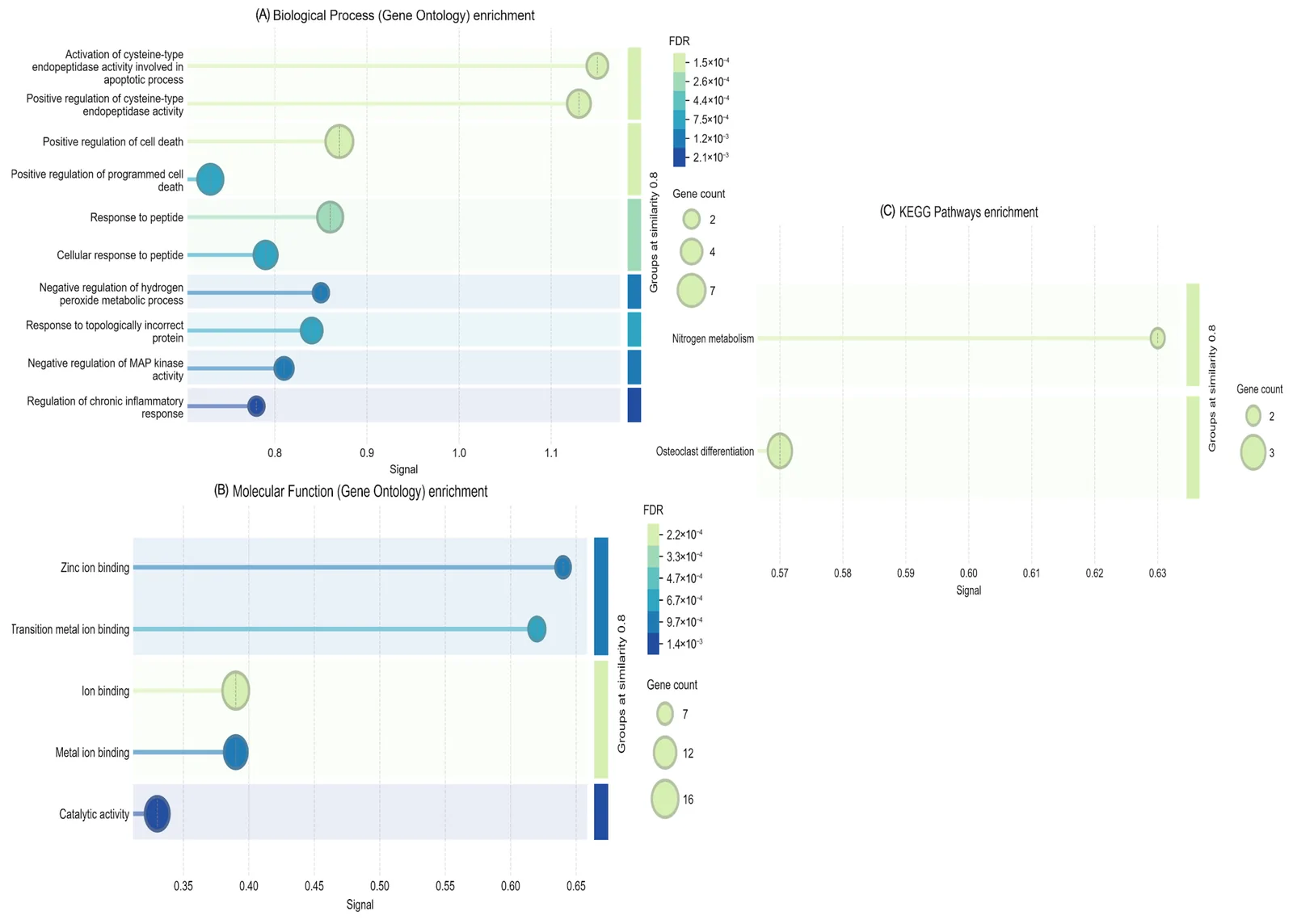
Gene Ontology (GO) and KEGG pathways analyses among the common genes. (**A**) Biological processes, (**B**) Molecular functions, and (**C**) KEGG pathways.

**Table 1:** Primer sequences.

Gene	Forward Primer	Reverse Primer
*Erα*	ACAAGGGAAGTATGGCTATGGAAT	CATAGTCATTGCACACTGCACAGT
*Erβ*	TTACGAAGTGGGAATGGTGAAGT	CACAAGGCGGTACCCACATC
*CASP3*	CAGTATGCCGACAAGCTTGAAT	TCTGTTGCCACCTTTCGGTTA
*PIK3CA*	AGCAAATGAGGCGACCAGAT	AGCAGGGTTTAGAGGAGACAGAAA
*AKT1*	GCTCAGCCCACCCTTCAA	CTCATCAAAATACCTGGTGTCAGTCT
*CDH1*	ACAGCCCCGCCTTATGATT	TCGGAACCGCTTCCTTCA
*CDH2*	TGGGAATCCGACGAATGG	GCAGATCGGACCGGATACTG
*ACTB*	CCAACCGCGAGAAGATGA	CCAGAGGCGTACAGGGATAG

## Data Availability

The data presented in this study are available in publicly accessible databases: SwissTargetPrediction (RRID: SCR_023756); ChEMBL (BPA: CHEMBL418971; BHB: CHEMBL1162496); DrugBank (BPA: DB06973; BHB: DBMET03174); GeneCards; OMIM (OMIM: 167000); Comparative Toxicogenomics Database (CTD); and STRING database. The original contributions presented in this study are included in the article material. Further inquiries can be directed to the corresponding author.
